# Why are people with dengue dying? A scoping review of determinants for dengue mortality

**DOI:** 10.1186/s12879-015-1058-x

**Published:** 2015-07-30

**Authors:** Mabel Carabali, Libia Milena Hernandez, Maria Jose Arauz, Luis Angel Villar, Valéry Ridde

**Affiliations:** 1 International Vaccine Institute, Dengue Vaccine Initiative, SNU Research Park, San 4-8, Nakseongdae-dong, Seoul, Gwanak-gu 151-919 South Korea; 2 Centro de Atencion y Diagnostico de Enfermedades Infecciosas (CDI), Bucaramanga, Santander Colombia; 3 grid.14848.310000000122923357School of Public Health (ESPUM), University of Montreal, Montreal, Quebec Canada; 4 grid.411595.d0000000121057207Universidad Industrial de Santander, Bucaramanga, Santander Colombia; 5 University of Montreal Public Health Research Institute (IRSPUM), Montreal, Quebec Canada

**Keywords:** Dengue, Dengue mortality, Social determinants of health, Scoping review

## Abstract

**Background:**

Dengue is a viral disease whose clinical spectrum ranges from unapparent to severe forms and fatal outcomes. Although dengue death is 99 % avoidable, every year around 20,000 deaths are estimated to occur in more than 100 countries. We consider that, along with biological factors, social determinants of health (SDHs) are related to dengue deaths as well.

**Methods:**

A scoping review was conducted to explore what has been written about the role of SDHs in dengue mortality. The inclusion criteria were that documents (grey or peer-reviewed) had to include information about dengue fatal cases in humans and be published between 1997 and 2013 and written in English, Spanish, Portuguese or French. The search was conducted using a set of key words related to dengue mortality in several electronic databases: PubMed, LILACS, COCHRANE, Scielo, Science Direct, WHOLIS, OpenGrey, OpenSingle and Google Scholar. Information on SDHs was categorized under individual, social and environmental, and health systems dimensions. A summative content analysis using QDA Miner was conducted to assess the frequency of information on SDHs and its contextual meaning in the reviewed literature. The role of each SDH in dengue mortality was assessed using content analysis results.

**Results:**

From a total of 971 documents retrieved, 78 met the criteria. Those documents were published in the Americas region (50.0 %), Asia (38.4 %), Europe (9.0 %) and Africa (2.6 %). The described SDHs related to dengue deaths included, in the individual dimension: age, ethnicity, education, type of infection and immunological status; and in the social dimension: poverty and care-seeking behavior. The health systems dimension included access, opportunity, and quality of care, as well as health staff knowledge. Ethnicity was considered a determinant that depends on cultural and socioeconomic conditions.

**Conclusions:**

Along with biological factors, there are several SDHs related to dengue mortality. However, only a few of these have been systematically analyzed, suggesting the need for more studies on this subject to inform the design and implementation of sustainable interventions to decrease dengue mortality. These findings nevertheless provide a better understanding of the non-biological factors involved in dengue mortality.

**Electronic supplementary material:**

The online version of this article (doi:10.1186/s12879-015-1058-x) contains supplementary material, which is available to authorized users.

## Background

Dengue is one of the most rapidly spread vector borne diseases and a major viral disease worldwide [1]. Dengue infection is caused by the transmission to humans of one of the four dengue virus serotypes (DENV1, DENV2, DENV3 and DENV4) through the bite of *Aedes* mosquitoes [2]. Over the past 50 years, dengue incidence has increased dramatically. Every year, around 100 million new cases are estimated to occur in 100–125 countries [1, 3]. Burdens of 96 million apparent and 293 million unapparent cases of the disease were estimated in 2010 [3, 4]. However, the real number of cases could not be identified due to the under/over-reporting or misdiagnosis of cases [4, 5]. Therefore, in the absence of any available vaccine, treatment, or effective vector control strategies, dengue remains a challenge for public health authorities worldwide [6, 7].

The clinical spectrum of this disease ranges from unapparent or asymptomatic to severe forms and fatal outcomes [8, 9]. The disease is characterized by the presence of fever, frontal headache, myalgia, arthralgia and cutaneous rash, usually self-limited to one week. Mild or asymptomatic infections are often associated with primary infections. Severe forms are characterized by the presence of hemorrhages, hypotension, thrombocytopenia and plasma leakage, also accompanied by neurological alterations [10], conditions that could eventually lead to shock and multi-systemic failure and that could worsen in presence of comorbidities [1, 8, 9]. In contexts where it is not possible to provide appropriate case management (because of limited resources, misdiagnosis or lack of knowledge), this could translate to fatal outcomes [1, 3, 8, 9]. Though dengue mortality is said to be 99 % preventable, case fatality rates (CFR) far higher than 1 % have been observed worldwide [1, 6, 11, 12].

Aside from intrinsic issues, foremost of which is virus infection, there are several other factors to which the increased disease incidence has been attributed. Uncontrolled urbanization, climate change and limited resources are some of the most important macro factors in this regard [1, 13–15]. There are also other elements, referred to as social determinants of health (SDHs), which are individual, social or health systems related factors that influence the health status of individuals and society [16–18]. Determined as well by the socioeconomic or political context, SDHs play an important role in the presence and development of several diseases, as described in the 2008 final report of the World Health Organization (WHO) Commission on Social Determinants of Health (CSDH) [18]. The SDHs generally described as related to dengue are water resources, sanitation, poverty and migration [19]. Ethnicity, gender and capacity to pay for health services are also known to play a role in the presence and management of dengue disease [14, 19–21]. However, information on the role that SDHs might play in dengue mortality is very limited.

We consider that SDHs (individual, social and/or health system related), along with the virus infection and the host conditions themselves, are related to dengue mortality. We therefore conducted a scoping review of the available literature on dengue mortality and its determinants to learn what has been described about this topic.

## Methods

A scoping review was conducted following the framework proposed by Arksey and O’Malley (2005) [22].

### Research questions

1. What has been described about SDH and dengue mortality?
2. What SDHs have been put forward as determinants for dengue mortality?

### Study design and search strategy

We conducted a scoping review of available literature using electronic databases (PubMed, LILACS, PAHO, MedCarib, COCHRANE, Scielo, Science Direct, WHOLIS, OpenGrey, OpenSingle and Google Scholar). Key words were: dengue/dengue fever/dengue hemorrhagic/DF/DHF/DSS, mortality, fatal cases/outcome, case fatality rate, CFR, determinants, social determinants and associated factors. Both MeSH (Medical Subject Headings) terms and free-text terms were used.

### Inclusion criteria

All documents in the published or grey literatures containing a report, description or analysis of fatal dengue cases in humans, and written in English, Spanish, Portuguese or French, from January 1997 to December 2013, were included. This time period was selected because the previous WHO dengue classification guidelines were published in 1997 and were valid until 2009, when the new guidelines became available [23].

### Screening

After the first filter from the electronic databases, the references were saved in an EndNote**®** library and were reviewed to identify potentially relevant papers. Those papers were assessed to determine whether they met the criteria and were then saved as potential documents. Additional sources were obtained after screening by crosschecking the references of previously identified papers.

### Data extraction

The documents selected based on the inclusion criteria were then assessed independently by two reviewers; as agreement between reviewers was >95 %, the team then captured the information using the data extraction form. That form was created using Epi Info 7™ and consisted of a spreadsheet to capture general information from each document, as well as STROBE checklist items [24]. Specific information such as dengue diagnosis, classification, fatal outcomes information and SDH components was also included. Although the documents were subjected to a quality assessment, all eligible documents were included in the review, regardless of the results of that assessment (Additional file 1).

### Data synthesis and analysis

General and specific information was summarized descriptively to chart the available literature (Additional file 2) . Documents were grouped according to the dengue classification used and SDHs [16] were categorized under three dimensions:

1. The *individual dimension*, which included characteristics such as occupation, income and education, as well as a subcategory labeled ‘biological component of the individual dimension’, which comprises age, sex, comorbidities, ethnicity and host conditions such as immunological status and type of infection (primary or secondary and severe or unusual forms).
2. The *social and environmental dimension*, which included social subcomponent aspects such as socioeconomic and political context, war and conflict, and social behavior. In the environmental component, humidity or rain seasonality/rainfall and geographical aspects were considered. Likewise, under the environmental component, we created a subcategory labeled ‘biological component of the environmental dimension’ to capture information about vector presence and intrinsic virus characteristics (i.e., identification of determinate serotypes or strain virulence).
3. The *health systems dimension*, which included information about health care access, coverage, opportunity and quality, as well as surveillance information.

To conduct a summative content analysis [25], the gathered data was migrated to QDA Miner (Provalis Research, Montreal, Canada), where each document was considered as a case and three key word categories were created for SDHs: ‘Individual’, ‘Social and Environmental’ and ‘Health Systems’. The summative content analysis, performed first, consisted of a manifest content analysis, for which the SIMSTAT feature was used to describe frequency of SDH key words by case and in all the reviewed literature. Second, a latent content analysis was performed, in which the WORDSTAT dictionary and the documents’ content were used to assess the contextual meaning of SDH key words in each case and among the reviewed documents. Using the results of the content analysis, each SDH’s role in dengue mortality was then also assessed. Both the ‘individual’ and ‘social and environmental’ dimensions included a subcategory for biological determinants to avoid dissociating SDHs from biological factors that might play a role in dengue fatal outcomes.

## Results

**Fig. 1:**
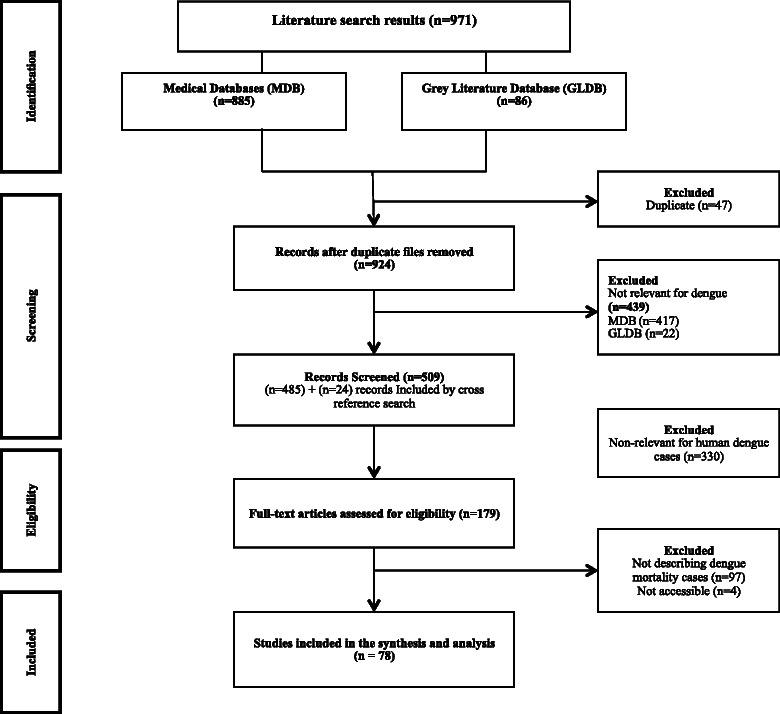
Flow diagram of studies selected. Multi Database (MDB) included the following databases: PubMed (*n* = 144), ScienceDirect (*n* = 566), Scielo (*n* = 69) and VHL, covering LILACS, PAHO, MedCarib, WHOLIS, and COCHRANE-CENTRAL (*n* =10). Grey Literature database (GLDB) included the following databases: Social Care Online, National Institute for Health and Clinical Excellence (NICE), System for information on Grey Literature in Europe (OpenSigle), National Guideline Clearing House, Health Development Agency, National Institutes of Health, Research Service Delivery and Organization Program (SDO), Research Register for Social Care, Google Scholar and OpenGrey (the last two specifically for grey literature in Spanish or other languages)

A total of 971 documents were retrieved, of which 179 were eligible and 78 were included in the review. Figure 1 describes the flow of the review.

### Geographical distribution, year and type of publications

**Fig. 2:**
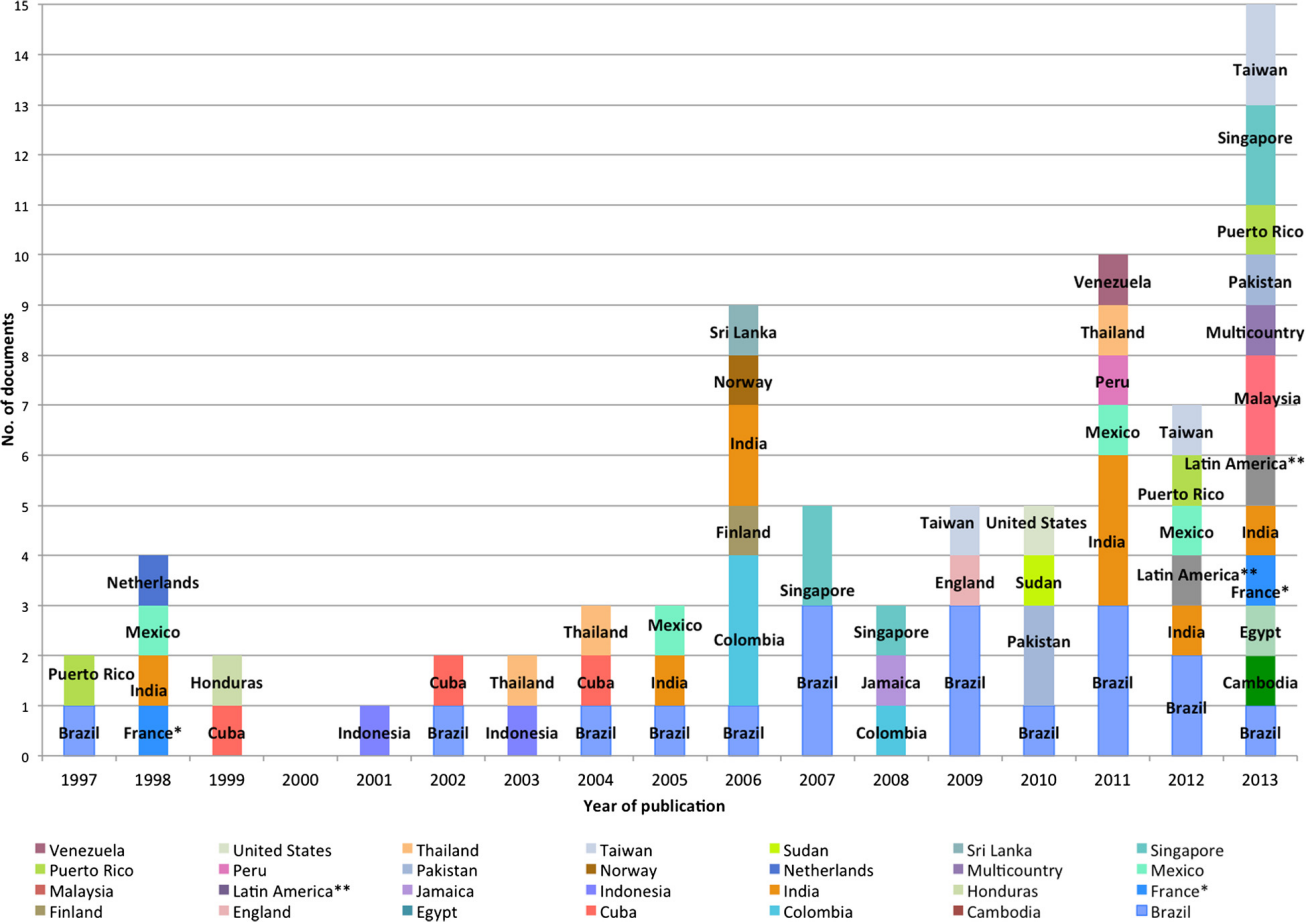
Distribution of documents included in the review by year of publication and country. *Make reference to Martinique; **Make reference to documents in which more than two countries in Latin America and the Caribbean region were described as the study site

The documents reviewed had a worldwide distribution. Half were published in the Americas region (*n* = 39/78), with Brazil representing 46.2 % (*n* = 18/39) of the papers in the Americas and 23 % (*n* = 18/78) of the total reviewed documents. The Asia, Europe and Africa regions followed, with 38.4 %, 9.0 % and 2.6 % respectively. There was an observed increasing trend in publications from 1997 to 2013 (Fig. 2).

Of the 78 documents reviewed, 60 (76.9 %) were descriptive, including single-patient or case series reports and ecological studies; 12 (15 %) were analytical studies, including cohort, case–control and cross sectional studies; and six (7.7 %) were review articles. The main sources used to conduct studies were hospital records (56.4 %), followed by a mix of surveillance data and hospital records (33.3 %) (Table 1).

**Table 1:** Characteristics of studies reviewed

Characteristics	n (%)
**Number of reviewed studies**	78
**Total no. of individuals included in the studies**	
Described number of dengue cases	1,900,499
Described number of fatal cases	8,650
**Region of publication**	
Americas	39 (50)
Asia	30 (38.4)
Europe	7 (9.0)
Africa	2 (2.6)
**Dengue case classification used**	
WHO 1997	64 (82)
WHO 2009	6 (7.7)
Other^a^	8 (10.3)
**Type of study**	
Descriptive	60 (76.9)
Analytical	12 (15.4)
Review	6 (7.7)
**Source of information**	
Surveillance data	3 (3.8)
Articles (for review documents)	5 (6.5)
Hospital chart and surveillance	26 (33.3)
Hospital charts	44 (56.4)
Study setting	
Hospital based	43 (55.1)
Population based	27 (34.6)
Other	8 (10.3)
**Primary outcome**	
General description of dengue cases	55 (70.5)
Specific description of dengue fatal cases	23 (29.5)
**Described cause of death**	
Only dengue	62 (79.5)
Dengue and other conditions	16 (20.5)
**Overall study quality** ^b^	
Well conducted	40 (51.3)
Could be improved	38 (48.7)

^a^Dengue cases classification defined by the document’s authors and does not correspond to official WHO guidelines

^b^Quality assessment performed according to STROBE checklist (http://www.strobe-statement.org)

### Dengue and dengue mortality

In 82 % of the documents, WHO 1997 [26] guidelines were used for classifying dengue diagnosis and dengue fatal cases, while 7.7 % used WHO 2009 guidelines [23]. The remaining 10.3 % used other classifications, such as Ministry of Health (MoH) adaptations of either the WHO 1997 or WHO 2009 guidelines, or the authors’ own classifications constructed on the basis of clinical, laboratory or pathological findings. Cause of death was attributed exclusively to dengue in 79.5 % (*n* = 62) of the papers and to dengue and other conditions in 20.5 % (*n* = 16).

### SDHs described in dengue mortality literature

**Fig. 3:**
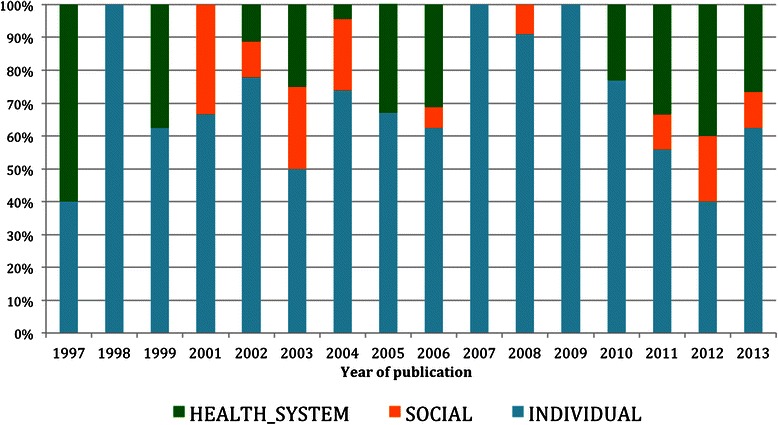
Distribution of the SDH key words set in the reviewed documents by year of publication

Content information related to the individual dimension was found in 88.5 % of the documents, health systems in 33.3 % and the social and environmental dimension in 28.2 %. Distribution of the SDH key words by year of publication is presented in Fig. 3. Comprehensive detailed information about the manifest content analysis is provided in Table 2.

**Table 2:** Frequency of information about individual, social and health systems dimension of SDHs

Characteristics	Presence of information	
	Yes (%)	No (%)
**Individual dimension**		
Age	75 (96.2)	3 (3.8)
Comorbidities	30 (38.5)	48 (61.5)
Type of infection	40 (51.3)	38 (48.7)
Immunological status	14 (17.9)	64 (82.1)
Sex/Gender	67 (85.9)	11 (14.1)
Ethnicity	17 (21.8)	61 (78.2)
Occupation	3 (3.8)	75 (96.2)
Income	1 (1.3)	77 (98.7)
Education	3 (3.8)	75 (96.2)
**Social and environmental dimension**		
Political context	3 (3.8)	75 (96.2)
War or Conflict		78 (100)
Poverty	2 (2.6)	76 (97.4)
Social Behavior	4 (5.1)	74 (94.9)
Environmental - Vector Presence	4 (5.1)	74 (94.9)
Virus characteristics	25 (32.1)	53 (67.9)
**Health system related**		
Specifications about health System	13 (16.7)	65 (83.3)
Access to health care	6 (7.7)	72 (92.3)
Health coverage	2 (2.6)	76 (97.4)
Opportunity for receiving attention	9 (11.5)	69 (88.5)
Quality of attention received	4 (5.1)	74 (94.9)
Length of hospital stay	10 (12.8)	68 (87.2)
Health staff knowledge	11 (14.1)	67 (85.9)
Surveillance	9 (11.5)	69 (88.5)

### Individual dimension

#### Biological components of the individual dimension

##### Age

Individuals’ chronological ages (in years or months) were described as relative or absolute frequencies of fatal dengue cases. There were more documents (*n* = 11) referring to high frequency of dengue deaths in adults [27–37] than in children (*n* = 5) [31, 38–41]. In two documents, age was not considered a determinant [42, 43].

##### Comorbidities

These were presented either as known pre-existing conditions, such as cardiac disorders, renal transplants, diabetes, hypertension, use of anti-platelet drugs, or pregnancy and pregnancy-related conditions [29, 30, 43–51], or as diseases confirmed during the course of dengue infection, such as concurrent bacterial infections, malaria, or other diseases [44, 52–56]. These comorbidity conditions were merely described and not explicitly reported as determinants for dengue deaths.

##### Type of infection and immunological status

Fatal cases were reported to be more common in patients with secondary infections, dengue hemorrhagic fever (DHF), dengue shock syndrome (DSS) or severe dengue than in those with dengue fever (DF) or dengue without warning signs [29, 34, 46, 55, 57–62]. There was also mention that dengue mortality was greater in cases with unusual manifestations [63, 64]. No deaths were mentioned in suspected primary infections.

##### Sex/Gender

The terms sex and gender were used without distinction in all reviewed documents. Information ruling out the role of sex as a dengue mortality determinant was often observed [45, 47, 52, 59, 63, 65–72]. Greater frequency of mortality in men was reported in 18 papers [35, 37, 39, 41–43, 55, 64, 73–81], whereas seven reported higher frequencies in women [27, 29, 30, 32, 58, 82, 83]. There was no description of significant differences between sexes in the presentation of fatal cases.

##### Ethnicity

This was reported either as the ethnic group to which individuals belonged or by mentioning their country of origin or self-assessment of their ethnicity. Descriptions included: Indians [30, 80, 84], Chinese [30, 54, 80, 85, 86], Malay [30, 80], Bangladeshi [30], African ancestry/‘Blacks’ or brown [27, 29, 35, 37, 87, 88], ‘Whites’ [27, 29, 35, 37, 82, 89, 90], and ‘Mixed’ [29, 37, 39]. Some papers described equivalent risk among all ethnic groups [52, 54, 60, 91], whereas others noted a protective role among Africans or persons of African ancestry [87], higher risk for severe forms among Whites [37], or that being Black or of African ancestry was determinant for dengue mortality [88].

#### Non-biological components of the individual dimension

##### Occupation

Described as either work status or activities performed, occupation was most often presented in the single or series case reports. Those whose occupation was defined by activities included students, housewives, army members, and non-qualified workers, among others [32, 42, 58].

##### Income

This component was not mentioned at the individual level, but was presented only as a variable of the Human Development Index (HDI) at the country or regional level [92].

##### Education

Only a few documents described education, usually by reporting the educational profile of the cases [32, 35]. One document showed an association between low education and dengue mortality [88].

### Social and environmental dimension

#### Socioeconomic and political context

Two papers discussed the socioeconomic and political context by describing dengue as a political issue [93] and the development of the disease in a city considered to be an economic hub [27].

#### Poverty

This was either described directly as the society’s economic situation or expressed as the HDI. Poverty was reported as a partial explanation for dengue deaths [87], and high CFRs were reported in low HDI countries [92].

#### Social behavior

The absence or delay of care-seeking was presented as an explanation for severe/fatal outcomes [77]. Dengue death was described as occurring more often in patients who sought care after the fourth or fifth day of fever, whereas those who recovered had generally sought care during the first three days [29–31, 76, 80, 93]. CFR decrease was attributed to increased awareness of dengue disease [91].

#### Environmental

Conditions such as humidity or rain seasonality/rainfall were related with the occurrence of outbreaks [27, 92, 94, 95]. Geographical barriers (e.g. distance or transportation constraints on access to health services) were not considered determinants of dengue mortality [93]. In one paper, rural residence was associated with higher probability of death by severe dengue [88].

#### Biological components of the environmental dimension

##### Vector presence

Increased dispersion of the vector, vector control difficulties, urban growth and increasing population mobility were important conditions determining vector and disease presence [87, 94, 96, 97].

##### Virus characteristics

Described either by the specific causal serotype, the serotype virulence or the effect of serotype combination, virus characteristics were present in 25 reviewed documents describing fatal cases [11, 29, 32, 33, 37–41, 52, 55, 60, 62, 66, 70, 73, 76, 79, 80, 83, 88, 98–101].

### Health systems dimension

#### Access

Papers described facilities where patients were managed (primary, secondary or tertiary level of care) or type of service accessed (private or public) [39, 69, 77]. Also described were barriers to health care access, including cases where patients died during the referral process, upon arrival at the facility, or after visiting a health facility outside of office hours [30–32, 40, 61, 65, 77].

#### Health coverage

An increase in population health coverage due to increased government health expenditure (GHE) was described as playing a determinant role in reducing dengue mortality. In some cases, over-representation of the private sector was related to limitations in disease management and consequent worse outcomes [92, 94].

#### Opportunity

Opportunity is defined as the condition in which health care is provided to the patient at the appropriate time. In nine articles, difficulties of opportunity were noted in diagnosis, case management and the referral process [30–32, 40, 65, 69, 88, 93, 102].

#### Quality of care

Qualitative descriptions of health care were presented in six documents. Descriptions covered good praxis, misdiagnosis, mismanagement (by early discharge, non-correction of shock, non-use of lab tests), or ‘wrong praxis’ related to both scientific and technical qualities [32, 40, 65, 69, 77, 93, 102].

#### Duration of hospitalization

This was reported as total number of days and/or average length of hospital stay in days, rather than as an association between length of stay and health outcomes [11, 69, 103]. Some documents described a rapid occurrence of death (at arrival or within the first 24 h) [30, 32, 40, 65, 80]. Others showed that death often occurred only after the third day, with hospitalizations sometimes extending more than 20 days [29, 39, 80].

#### Health staff knowledge

This was described as the presence of any knowledge about diagnosis or cases management and the quality of that knowledge (i.e., adequate or inadequate). Some papers noted that adequate or improved patient management by health staff had a positive effect in terms of declining dengue mortality [32, 51, 65, 91, 93, 102].

#### Surveillance

This was reported as the presence or absence of a surveillance system in the study settings. The need for systematic and integrated surveillance to improve health care services was often noted [32, 51, 69, 91, 92, 94].

In summary, according to the reviewed literature, the SDHs considered to be determinants for dengue mortality were: age, type of infection, ethnicity, education, poverty, care-seeking behavior, access, opportunity and quality of care, and health staff knowledge. The latent content analysis is described in Tables 3, 4 and 5.

**Table 3:** Individual dimension aspects related to dengue mortality according to content analysis

Individual dimension	Consideration as determinant for dengue mortality		References
SDH	Concept^a^	Observations	
Age	Yes	• Age was more often described as a determinant than not.	[27–43]
		• In children the most affected group were those <15 years old with an emphasis on the group of <5 years old.	
		• A higher frequency of dengue mortality was reported in adults (mostly in the Americas region)	
		• Determinant related with immunological status, type of infection and comorbidities.	
Comorbidities/ Pre-existing conditions	NC	• Although these conditions might worsen the dengue status, there were not described as directly related to fatal outcomes.	[29, 30, 43–56]
		• Overlaps of diseases make differential diagnosis difficult and could be considered as independent causes of death.	
		• The most cited were diabetes, bacterial infections and pregnancy.	
Infection type/Immunological status	Yes	• Secondary infections, severe forms and unusual presentations were described as determinants.	[29, 34, 46, 55, 57–64]
Sex/Gender	NC	• More dengue cases were described in women, even though the majority of dengue deaths were reported in men.	[27, 29, 30, 35, 37, 39, 41–43, 45, 47, 52, 55, 58, 59, 63–83]
		• Statistically significant differences were described between sexes in dengue in severity but not in mortality.	
		• Gender differences in frequency were related to care-seeking behavior patterns.	
Ethnicity	Yes	• A protective role was described for African ancestry/‘Blacks’ and a risk factor for Caucasian/ ‘Whites’.	[27, 29, 30, 35, 37, 39, 52, 54, 60, 80, 84–91]
		• Described also as determinant related to socioeconomic status and cultural behaviors.	
Occupation	No	• Some occupations were listed (mostly in the single case reports) but none was directly linked to fatal outcome.	[32, 42, 58]
Income	No	• There were no individual reports on the fatal cases’ income.	[92]
		• Although it is related to socioeconomic status, income was not reported as a determinant for dengue mortality.	
Education	Yes	• Level of education was described in some cases.	[32, 35, 88]
		• In the content analysis, it was observed that education was described as a determinant for dengue mortality related to knowledge of patients and health staff.	

^a^Concept according to what was described in the literature. *YES* Considered to be a determinant, *NO* Not considered to be a determinant, *NC* Non-conclusive information

**Table 4:** Social and environmental dimension aspects related to dengue mortality according to content analysis

Social and environmental dimension	Consideration as determinant for dengue mortality		References
SDH	Concept^a^	Observations	
Poverty	Yes	• Contributes directly to income, education and living conditions.	[87, 92]
		• Represents a barrier for access to health care and thereby contributes to rise in dengue mortality.	
Social behavior	Yes	• Absence or delays in care-seeking were described as explaining dengue mortality.	[29–31, 76, 77, 80, 91, 93]
		• Reflects social and cultural aspects and is related to risk perception and awareness of disease by patients and health staff.	
Environmental/vector presence	NC	• Rural residence and geographical barriers were more related to access to health care than to dengue mortality itself.	[27, 87, 88, 92–97]
		• Vector presence and occurrence of dengue outbreaks were described as a condition that increased the risk of severe forms of dengue.	
Virus characteristics	Yes	• Heterogeneous infections and virulence of strains might increase disease severity	[11, 29, 32, 33, 37–41, 52, 55, 60, 62, 66, 70, 73, 76, 79, 80, 83, 88, 98–101]
		• Together with type of infection/immunological status, was described as an important determinant.	

^a^Concept according to what has been described in the literature. *YES* Considered to be a determinant, *NO* Not considered to be a determinant, *NC* Non-conclusive information

**Table 5:** Health systems dimension aspects related to dengue mortality according to content analysis

Health systems dimension	Consideration as determinant for dengue mortality		References
SDH	Concept^a^	Observations	
Access to health care	Yes	• Lack of access or presence of barriers to health care and/or supplementary services were often	[30–32, 39, 40, 61, 65, 69, 77]
		• described in dengue mortality.	
Health coverage	NC	• Although health coverage might facilitate the access and health care attention received, it was not specifically described as determinant.	[92, 94]
Opportunity for receiving attention	Yes	• Limitation in referrals, shock corrections, delayed attention, or early hospital discharge were the most described aspects related to opportunity for attention.	[30–32, 40, 65, 69, 88, 93, 102]
Quality of attention received	Yes	• Related as well to health staff knowledge, both technical and scientific quality of attention were described as direct determinants in the cases with wrong praxis.	[32, 40, 65, 69, 77, 93, 102]
Length of hospital stay	NC	• This item was reported by describing the duration of a hospital stay, but without reporting any association with fatal outcomes.	[11, 29, 30, 32, 39, 40, 65, 69, 80, 103]
Health staff knowledge	Yes	• Expressed as appropriate management of the disease, thereby decreasing the chance of developing severe forms and dengue mortality.	[32, 51, 65, 91, 93, 102]
Surveillance	No	• Described as a tool for cases analysis and documentation of outbreaks but not as a determinant.	[32, 51, 69, 91, 92, 94]

^a^Concept according to what has been described in the literature. *YES* Considered to be a determinant *NO* Not considered to be a determinant, *NC* Non conclusive information

## Discussion

This scoping review provides detailed information on the social determinants of health in dengue fatal outcomes. From an extensive exploration of the literature, this review compiled 16 years of worldwide data providing valuable information to analyze the role of SDHs in dengue mortality in endemic countries. While there are reviews exploring determinants of mortality, these have been mainly regionally and biomedically oriented [11, 29, 55, 80, 88, 92]. This is the first review, to our knowledge, to explore the role of SHDs as determinants for dengue mortality with a global scope.

Different types of documents were reviewed, with different study designs, outcomes and sources. However, there was a notable lack of qualitative or social sciences oriented studies. This might be due to the fact that available information about dengue cases is usually focused on biological factors and the available evidence is published in biomedical databases [88, 93]. Nonetheless, even if SDHs are known to play an important role in people’s health [19, 104, 105], the lack of literature on the links between SDHs and specific health outcomes is understandable. It may be more feasible and suitable to find associations between biological variables and biological outcomes, which are both easier to understand and to prove, and which is not the case for non-biological factors [18, 19, 106]. Analyzing SDHs is a very complex undertaking, and in the case of multifactorial neglected tropical diseases (NTDs) like dengue, exploring interactions among results is far more complicated [19]. There were only two articles specifically addressing the topic in this review, one discussing the role of the health system using a qualitative approach [93] and the other, with a quantitative approach, exploring the role of social determinants in dengue mortality using a national surveillance dataset [88]. Still, there is currently an interest in exploring the relationship between SDHs and health [19, 104–106], and this type of review has much to contribute in this area.

We expected to find more documents from the Asia region due to its large dengue presence. These sparse results may be due to the fact that CFRs there decreased after the 1980s [1, 10, 107], such that there was less reported dengue mortality during the time covered by the review. In contrast, CFRs in the Americas region have increased significantly over recent years [5, 11, 15, 107]. The African region, on the other hand, was a special case. Obtaining information about dengue in African countries remains a challenge, and very little is known about severe cases and mortality [5, 108]. In this review, while there were few articles on Africa, those were very valuable, showing an emergent interest in dengue research in Africa. Nonetheless, under-reporting and difficulties associated with attributing dengue as the cause of death are reflected in the limited available information overall [3–5]. Therefore, given the nature of the topic and the heterogeneity of the documents found, we felt that a scoping review including content analysis was a suitable methodology that would allow us to better analyze and understand the findings.

### Social determinants of health related to dengue mortality

Age was described as a determinant related to immunological status and type of infection [1, 8, 9, 107]. Interestingly, despite the fact that reported mortality rates were higher in adults, children’s cases seemed to be more sensitive from a sociocultural perspective and generated greater concern. Moreover, fatal outcomes in children were described as also being dependent on health staff and parents’ awareness of the disease, implying that children’s outcomes depend not only on age, but also on adequate management by health staff and opportune care-seeking behavior by parents [29, 37, 38, 93, 107]. Likewise, ethnicity was considered to be a determinant for dengue mortality despite the known biological implications. For instance, while some papers have posited African ancestry or being ‘Black’ as a protective factor for severe forms and dengue mortality [107, 109–112], this review found a paper from Brazil contradicting that position [88]. We observed that when the protective role of African ancestry was demonstrated in other studies, socioeconomic information was not taken into account, and that when this information was considered as a possible risk factor, the social aspect appeared as a co-determinant. This could be related to the fact that people of African ancestry in the Americas tend to live in unfavorable socioeconomic conditions [88]. Hence, it is not the fact of being ‘Black’ in Brazil that increases the chances of dying from dengue, but rather living in unfavorable socioeconomic conditions, as presented by Blanton et al. in 2008 [109]. When both ethnicity and income were addressed, and even after controlling for income, African ancestry (both self-reported and genotyped) was a protective factor for severe forms of dengue. As such, ethnicity would not be a determinant on its own but is linked with socioeconomic position and opportunity for access to qualified services [18, 19, 88].

Education is a determinant with a twofold effect on dengue mortality. First, educated people will understand the importance of the disease and its risks, seek care opportunely and adhere to treatment [1, 17, 88]. Second, educated health staff will manage patients accurately, thereby decreasing their chances of developing severe forms, which in turn reduces dengue mortality [32, 91, 93, 107]. Furthermore, the level of education (and awareness) of individuals could be a proxy for poverty (and poor quality of facilities), since it is related to the quality of life of individuals and societies and to the quality of the health system [16–19].

Type of dengue infection, virus characteristics and immunological status of the individual are known biological determinants of severe forms and fatal outcomes of dengue [3, 6, 8, 9, 13, 33, 37, 67, 107, 110, 113]. However, it is known that individuals’ immunological status is also related to socioeconomic status (SES) [19, 104, 105]. Moreover, in the case of dengue, type of infection and immunological status will depend on previous exposure to the virus. Acting as a selective pressure, previous exposure induces changes in the virus and allows it to remain in a determinate region, leading to endemicity [1, 6, 7, 9, 19, 23]. As such, those determinants, along with their biological implications, could also be considered an outcome of the socioeconomic and environmental conditions in which people are living.

Poverty and social behavior were considered to be general (wide-ranging) social dimension determinants for dengue mortality, related to income, education and health systems. Lack of economic resources is often associated with the presence of dengue [1, 6, 7, 19, 109]. Poverty will also have an impact on access to health care, since in many developing countries access is determined by capacity to pay [16, 18]. Likewise, poverty could limit access to care in remote areas [17–19]. It is also known that lack of economic resources may lead to fatal outcomes and have devastating economic implications [11, 21, 87, 92, 94]. Furthermore, society is moved by cultural or idiosyncratic patterns, whether hierarchical, economic, power or gender-oriented, that affect people’s health status [16–19, 104]. Some documents in this review observed that care-seeking behavior may determine the possibility of receiving opportune treatment and thereby avoiding fatal outcomes [29, 30, 76, 77, 80, 93]. A relationship was also observed between care-seeking behavior patterns and gender, in accordance with societal cultural behaviors [18, 19, 35, 93, 94].

The health systems determinants related to dengue mortality were access to health care, opportunity and quality of care, and health staff knowledge. Indeed, health systems play a determinant role in the health outcomes of people, as mentioned by the CSDH [16–18]. Access to health care is nevertheless mediated by other SDHs, such as income and poverty [16, 18–20, 104, 114], and it is therefore essential to recognize that limited access to health care may contribute to the development of dengue fatal outcomes [3, 19, 21, 30–32, 40, 92]. In addition, opportunity and quality of care and health staff knowledge are determinants related to education in several ways. In the case of opportunity for care, empowered and disease-aware individuals tend to seek care opportunely [19] and knowledgeable staff tend to offer opportune management to dengue patients [18, 19]. When care is not offered opportunely, the risk of dengue fatal outcomes is theoretically doubled [27, 31, 55, 61, 80, 88, 93, 102]. As for quality of care and health staff knowledge, trained personnel translate into better case diagnosis and management, and consequently low mortality rates [32, 91, 93, 107, 114]. Therefore, the above-mentioned health systems related determinants must figure prominently in any strategy targeting a decrease in dengue mortality.

### Described SDHs non-related to dengue mortality

Although sex/gender was not considered a determinant for dengue mortality in this review, some authors observed that its role in fatal outcomes depended on care-seeking behavior, which in turn depended on sociocultural aspects [27, 88, 94]. As gender, being socially constructed, is differentiated from sex, and since gender roles interact in different forms with NTDs [18, 19], further research on this topic would be interesting. In the case of dengue, it might not be sex, but rather care-seeking behavior driven by gender, that determines the outcome [4, 88]. With regard to the presence of comorbidities or pre-existing conditions, the information provided in the reviewed documents was not specific and therefore not conclusive enough to consider it a determinant for dengue mortality. Nonetheless, it is important to acknowledge that comorbidities might aggravate the disease, as has already been stated [8, 12, 110]. Likewise, although income and occupation were not described as determinants, these two variables are related with socioeconomic status and will depend on education as well. Hence they are considered social determinants in relation to many infectious diseases, including dengue [18, 19, 109].

Very few documents provided information on environmental factors, and neither the few environmental factors described nor vector presence, were considered direct determinants for dengue mortality. Aspects such as urban residence or level of urbanization were reported only when describing dengue cases but not when informing about fatal cases. Nonetheless, vector presence could depend on virus presence and social behaviors (community or government actions). Moreover, vector control is the current feasible strategy for dengue control while waiting for vaccine development [1, 3, 6, 9, 13, 14]. Neither health care coverage nor hospital length of stay was conclusive as a determinant for dengue mortality. Opportunity for care by health staff might be related to coverage and access to health care, and hospital length of stay might be related to quality of care and health staff knowledge. These conditions, along with the presence of a surveillance system, are useful aspects to consider when analyzing the presence of dengue and its outcomes.

### Strengths and limitations

This review’s inclusiveness and the comprehensiveness of the analysis are major strengths. Including a great variety of documents and conducting a review in four languages helped reduce selection bias and increased the scope of the information in this type of review [22]. However, the fact that we did not include documents written in native languages from the Asia region (Thai, Vietnamese, Indonesian, etc.) could have limited the number of documents reviewed from that region. Likewise, it is possible that the Americas region was over-represented by the fact that the review included documents written in Spanish and Portuguese. A summative content analysis [25] of dengue mortality documents provided comprehensive and detailed information on the topic over the past 16 years. It is important to acknowledge that, despite the substantial dengue literature, information on fatal cases is still limited and is mostly of a biomedical nature. Moreover, access to some documents was not possible. An added challenge is the fact that it is not always possible to distinguish between SDHs and biological factors, nor to identify their determinant roles separately [18, 19, 104]. Notwithstanding the above-mentioned limitations, this scoping review offers valuable information on the role of SDHs in dengue mortality.

## Conclusions

Dengue is a multifactorial disease, and although there is much information on the presence of the disease, there is limited information on the causes of dengue mortality. These findings reveal that, along with biological factors, SDHs play an important role in dengue fatal outcomes. However, only a few of these determinants have been systematically analyzed. Moreover, findings show that even in the presence of available guidelines for disease management and strong commitment to dengue control, there are still many people dying from a 99 % avoidable cause of death, attesting to the need for more studies on the role of SDHs in dengue mortality. Studies that reveal the important SDHs involved in dengue mortality will help policy-makers and health practitioners build better and more sustainable interventions to reduce dengue mortality rates worldwide.

## Additional files

Additional file 1:
**Table S1.** Scoping Review dengue mortality and SDH database: Spreadsheet with the information from the reviewed documents. (XLSX 84 kb)
Additional file 2:
**Table S2.** PRISMA checklist. Format. (DOC 62 kb)

## Abbreviations

CFR: Case fatality rates
CSDH: Commission of social determinants of health
DENV: Dengue virus
DF: Dengue Fever
DHF: Dengue Hemorrhagic Fever
DSS: Dengue Shock Syndrome
HDI: Human Development Index
GHE: Government Health Expenditure
MeSH: Medical Subject Headings
MoH: Ministry of Health
NTD: Neglected tropical diseases
SDH: Social determinants of Health
SES: Socioeconomic status
WHO: World Health Organization
